# Fat talk, old talk, or both? Association of negative body talk with mental health, body dissatisfaction, and quality of life in men and women

**DOI:** 10.1186/s40337-023-00803-1

**Published:** 2023-05-18

**Authors:** Savannah C. Hooper, Lisa Smith Kilpela, Victory Ogubuike, Carolyn Black Becker

**Affiliations:** 1 Barshop Institute, UT Health San Antonio, 7411 John Smith Dr. Suite 1050, San Antonio, TX 78229 USA; 2 ReACH Center, UT Health San Antonio, San Antonio, TX USA; 3 South Texas VA Health System, Audie Murphy Veterans Hospital, San Antonio, TX USA; 4 Department of Psychology, Trinity University, San Antonio, TX USA

**Keywords:** Fat talk, Weight talk, Old talk, Body dissatisfaction, Eating disorder pathology, Quality of life

## Abstract

**Background:**

Little research has investigated the harmful effects of old talk—negative age-related body talk—on mental health and quality of life despite substantial research examining fat talk. Old talk also has only been evaluated in women and in relation to few outcomes. Of note, old talk and fat talk are strongly correlated, suggesting possible overlap in elements that drive negative outcomes. Thus, the primary aim of this study was to investigate the extent that old talk and fat talk contribute to negative mental health and quality of life outcomes when examined in the same model and when interacting with age.

**Methods:**

Adults (*N* = 773) ages 18–91 completed an online survey assessing eating disorder pathology, body dissatisfaction, depression, aging anxiety, general anxiety, quality of life, and demographics.

**Results:**

While fat talk and old talk were correlated with almost all outcome variables, fat talk was more commonly significantly associated with poorer outcomes than old talk. Additionally, the relationship between fat talk and old talk with poorer mental health was affected by age in men, but not women.

**Conclusions:**

Future research is warranted to decipher the individual effects of old talk and fat talk on mental health and quality of life across the adult lifespan.

## Abstract (summary)

The term “negative body talk” is used to describe the negative things people sometimes say about their bodies and appearance. Two kinds of negative body talk are fat talk (critical and negative talk about weight-related body image) and old talk (negative body talk focused on changes due to aging). Fat talk has been widely studied and found to be related to poorer mental health, including body dissatisfaction and eating disorders. However, old talk has been much less studied though it is both similar and distinct from fat talk. The current study examined how both old talk and fat talk related to mental health and quality of life in adults across the lifespan. We found that both were correlated with poorer mental health, but when compared to one another, fat talk was more related to mental health in both men and women. Of note, age appears to impact these relationships in men, but not in women. It is important for future research to examine why age seems to influence the impact old talk and fat talk have on mental health and further evaluate the similarities and differences between these two types of negative body talk.

## Background

It is well documented that body dissatisfaction is pervasive across the lifespan and is harmful to mental health [1, 2]. Body dissatisfaction prospectively predicts eating disorder (ED) symptoms, depressive symptoms, low self-esteem, and is associated with suicidal ideation in adolescent boys and girls [3–7]. It also is associated with anxiety, depression, psychological distress, and ED pathology in adult men [8–10] and greater restrained eating and bulimic symptoms, thin-ideal internalization, aging anxiety, and depression in midlife women [11, 12]. While fewer studies have examined body dissatisfaction in older adults, in a sample of women 60–70 years, over 60% reported body dissatisfaction [13]. Given both the pervasive nature of body dissatisfaction across all ages and the negative consequences, understanding how various factors promote or exacerbate this phenomenon are needed.

A growing body of evidence suggests that negative body talk, specifically ‘fat talk,’ contributes significantly to body dissatisfaction. Although originally used to describe the negative or critical body image talk in which pre- and adolescent girls engage regarding the shape and size of their bodies [14], individuals of all ages appear to engage in this behavior (e.g., [15]). Fat talk perpetuates Western appearance ideals (i.e., thin-ideal for feminine beauty; muscular ideal for male attractiveness) and includes phrases such as “I’m so fat” or can be seemingly positive, like “Wow, you look great! Did you lose weight?” Fat talk is associated with disordered eating behavior, depression, and is a risk factor for body dissatisfaction in men and women [16–19]. Experimental research indicates that even a few minutes of listening to fat talk can worsen state body image [20].

While fat talk perpetuates the Western notion that fatness is bad, it is important to note that these appearance ideals also promote youthfulness. Indeed, there is significant pressure for individuals, especially women, to conform to youthful appearance ideals or to age ‘gracefully’ as success and social acceptability are associated with a thin, young body [21–23]. For women, midlife and older adulthood bring a host of bodily changes that can shift women further from this thin-young ideal. Hormonal fluctuations, pregnancy, and menopause are all associated with weight gain; moreover, changes in body fat distribution, greying hair, skin wrinkles, and darks spots accompany aging [24]. Media messages also reinforce the notion that aging-related appearance changes are to be feared, with constant bombardment of anti-aging products and surgical procedures designed to slow or reverse the signs of aging [21]. Additionally, aging anxiety has been significantly associated with body surveillance, appearance control, and body image avoidance in college students and anti-aging behaviors in midlife women [23].

Thus, as pressures to remain thin often manifest in engaging in fat talk, pressures to maintain a youthful appearance may lead women to engage in negative *age-related* body talk, (colloquially known as ‘old talk,’ and referred to as such throughout the rest of this paper) [25]. Like fat talk, old talk includes negative or seemingly positive phrases about one’s body and appearance, such as “I have too many wrinkles,” “I wish I looked as young as them,” or “You look so good for your age!” However, in comparison to the more substantial fat talk literature, old talk is significantly understudied. Thus, the current study aims to fill in research gaps, investigating how old talk and fat talk interact with age to impact mental health across the lifespan.

To date, only three studies have examined negative age-related body talk. In 2013, Becker and colleagues conducted the first study examining old talk to determine its prevalence and relationship to fat talk, body image, ED pathology, and aging anxiety. They found that in a sample of adult women (*M* age = 36.8), 66% of women in their sample engaged in old talk. The older women in the sample engaged in more old talk than the younger women, though almost half of the younger age group engaged in at least occasional old talk. Old talk was significantly correlated with body image disturbance, ED pathology, and aging anxiety. The correlation between old talk and body image disturbance and ED pathology increased with women’s ages. Additionally, fat talk and old talk were significantly but not perfectly correlated, indicating they are related but separate constructs. In 2014, Arroyo and colleagues examined old talk in college aged women, finding that old talk mediated the relationship between self-objectification and body dissatisfaction, drive for thinness, and bulimia [26]. Additionally, Arroyo and Andersen [27] evaluated the effect of mothers' fat talk and old talk on their daughters’ negative body talk. Old talk was related to the mothers’ and daughters' own body image outcomes and mothers’ old talk was positively related to their daughters’ body dissatisfaction.

Despite these significant results, many questions remain as to the consequences of engaging in old talk, fat talk, or both. Of note, the only three existing studies examining old talk have important limitations. For example, each study exclusively surveyed women. However, men frequently report body dissatisfaction and engage in negative body talk [8, 17, 28]. Lasher and Faulkender [29] also found that men report more aging anxiety than women, which could lead to engaging in old talk. Therefore, research is warranted investigating negative age-related body talk in men.

Secondly, old talk has only been examined in relation to body image, aging anxiety, and ED pathology outcomes. However, fat talk and body dissatisfaction are associated with other mental health indicators, such as depression and general anxiety [8, 16, 22, 30]. Body dissatisfaction also has been associated with poorer quality of life (QOL; [31]), yet little to no research has examined the influence of engagement in fat talk on QOL and none regarding old talk, despite reasons to expect harmful effects.

Furthermore, not only have both old talk and fat talk been previously associated with poorer mental health, but the two constructs are strongly correlated. Thus, it is unclear whether old talk is related to poorer outcomes because of its similarities to fat talk or whether old talk is unique in its relation to mental health indicators. If old talk is unique and at least partially independent in its relations to body dissatisfaction and poor mental health outcomes, different intervention strategies targeting old talk to decrease body dissatisfaction may be necessary as current interventions targeting body dissatisfaction focus more on fat talk and combating the thin ideal. However, old talk is centered on both the ‘thin’ ideal and the ‘young’ ideal. If old talk indeed operates independently, this may have implications for body dissatisfaction interventions, suggesting more emphasis and discussion on accepting the aging process and the physical changes associated with aging are warranted.

Therefore, the primary aim of this study was to begin filling in these gaps in the literature by examining the association of engaging in fat talk and old talk with body dissatisfaction, ED pathology, depression, and other mental health and health-related QOL indicators in adult men and women. To begin examining potential overlap in these two constructs in relation to their impact on mental health, we placed fat talk and old talk in the same regression models as predictor variables. We also investigated how age interacts with these relationships, as fat talk is prevalent across the lifespan and old talk increases with age [25]. We predicted an age by old talk interaction, such that old talk would be significantly associated with all outcome variables for both men and women, with these relationships strengthening with age. We hypothesized that fat talk would be significantly related to all outcomes as well, but age would not influence these relationships as previous research suggests fat talk is pervasive and harmful across the lifespan [15, 25].

## Method

### Participants

The initial sample for this study was 833 participants. However, because our planned analyses (see below) were anchored on gender, participants who did not report their gender were excluded from the sample (*n* = 51). We also only received four responses from non-binary individuals. Therefore, due to insufficient data to examine gender minority responses independently in exploratory analyses (which we had hoped to conduct), we excluded these participants as well. The final sample included 778 adults (Men *n* = 288, Women *n* = 490) ages 18–91 years (*M* = 39.90, *SD* = 18.14). Of note, we chose to include older adolescents and young adults in this study rather than exclusively focusing on midlife and older adults, despite old talk being more common in midlife and older women. Becker et al. [25] found that almost half of their youngest age group (ages 18–29) engaged in old talk and Gendron and Lydecker [23] found that body image is related to aging anxiety in young adults. Due to the international social network of the research team, participants were recruited from the United States, the United Kingdom, and other Western European countries.^1^ In this sample, the racial/ethnic breakdown was 63.8% White/Caucasian, 18% Hispanic, 4.5% Asian, and 4.2% Black/African American (see Table 1). Regarding other demographics, 87.3% reported heterosexual sexual orientation, 61.1% reported having a bachelor’s degree or higher, and 52.2% reported being married or living with someone in partnership. We investigated gender differences in socio-demographic variables.^2^ Women in this sample were significantly older than the men, had a higher median education completion, and a greater portion of women were married compared to men. There were no gender differences in mean body mass index (BMI).

**Table 1:** Demographics and clinical characteristics by men and women

Measures	Men (*n* = 288) *M (SD)* or *N* (%)	Women (*n* = 490) *M (SD)* or *N* (%)
Age	37.91 (19.83)	41.08 (16.97)
Body Mass Index	26.81 (5.22)	27.09 (6.87)
*Race/ethnicity*		
White	180 (62.5%)	316 (64.5%)
Black or African American	9 (3.1%)	24 (4.9%)
Asian	11 (3.8%)	24 (4.9%)
Hispanic/Latino	58 (20.1%)	82 (16.7%)
Multiple races/other	30 (10.4%)	41 (8.3%)
*Education*		
Graduated high school or less	30 (10.7%)	24 (4.9%)
Some college	127 (44.1%)	119 (24.3%)
Bachelor’s degree	54 (18.8%)	161 (32.9%)
Some graduate school	19 (6.6%)	35 (7.1%)
Graduate school degree	56 (19.5%)	151 (30.8%)
*Relationship status*		
Married/living with partner	117 (40.6%)	264 (53.9%)
Single	154 (53.5%)	149 (30.4%)
Divorced/separated	14 (4.1%)	35 (7.1%)
Widowed	1 (0.3%)	14 (2.9%)
*Clinical cutoffs*		
Depression	69 (24%)	121 (24.7%)
General anxiety	63 (21.9%)	137 (28%)
ED behaviors*		
Binging	60 (20.8%)	101 (20.6%)
Vomiting	2 (0.7%)	10 (2.0%)
Laxative use	1 (0.3%)	17 (3.5%)
Restricting	83 (28.8%)	155 (31.6%)
Excessive exercise	86 (29.9%)	121 (24.7%)

ED Behaviors = disordered eating behaviors

*Disordered eating behaviors are behaviors participants reported engaging in once or more in the past week

### Procedures

This study received approval by the Institutional Review Board. Recruitment began in October of 2020 and ended in May of 2022. Participants were recruited through online social media platforms (e.g., Facebook groups, Instagram), flyers in local senior centers, clinics, coffee shops, and libraries, as well as personal and professional networks of the research team. Individuals recruited online were encouraged to forward the survey to their own social network or repost the survey on their social media. Throughout recruitment, careful monitoring of data revealed two small influxes of artificial responses (i.e., non-human “bots”), which is a common problem online survey studies encounter [32]. We discarded all artificial responses and embedded validation questions into the survey. Only responses from eligible participants that passed validity checks (e.g., invalid or nonsensical text, checks for unreasonably short completion time, repeated entries) were included in analyses. After consenting to participate, participants completed measures of frequency of engagement in fat talk and old talk, as well as measures of body dissatisfaction, ED pathology, depression, aging anxiety, general anxiety, QOL, and demographics. If participants chose to provide their email upon completing the survey, they were entered into a raffle to potentially win one of four $50 Amazon gift cards.

### Measures

#### Fat talk and old talk

To assess engagement in fat talk, participants completed the Negative Body Talk Scale (NBTS; [33]). This scale was originally developed to measure the likelihood of women to engage in negatively valanced commentary about their own body weight and shape when speaking with others. The NBTS includes 13 items, which create a body concerns subscale and a body comparison subscale. The researchers of the current study changed any female pronouns to “they” or “theirs” to make this scale gender neutral. For example, instead of “I wish my body looked like hers,” the new item became “I wish my body looked like theirs,” or from “She’s in such good shape,” to “They’re in such good shape.” This was the only modification made to the NBTS for the assessment of fat talk in this sample. The NBTS has been modified similarly in past research; Arroyo and Brunner [34] changed the pronouns to “she/him” or “hers/his.” However, because “they” is increasingly accepted as a singular gender-neutral pronoun, and because we wanted to be more gender inclusive, we used “they.” The original scale has strong evidence of discriminant validity, incremental validity, and internal consistency (α = 0.94; [33]). It also previously demonstrated moderate test–retest reliability across 4–6 weeks (*r*(43) = 0.74; [33]). Participants reported how frequently they may say a certain phrase on a 7-point Likert scale, ranging from “never” to “always.” Items are averaged for a total score. Internal consistency in this sample was excellent (Men: current α = 0.93; Women: α = 0.94).

Frequency of engagement in old talk was measured using a different modified version of the NBTS. This is not the Old Talk Scale that was created by Becker et al. [25], which was a very female-centric measure and provided scenarios where a character ‘Anna’ speaks with her friends and old talk arises. This scale would have required significant adaptations to be appropriate for the current study. In contrast, the NBTS needed markedly less modification to assess old talk. This was the rationale for modifying the NBTS in the present study. Scale items were modified to shift from a fat talk focus to an old talk focus. For example, instead of “I feel fat,” the new item became “I feel old,” or from “I need to go on a diet” to “I need to look younger” (see “Appendix”). We also used the same gender-neutral pronoun as for fat talk. The new scale remained 13 items; participants reported how frequently they may say a certain phrase on a 7-point Likert scale, ranging from “never” to “always.” Items are averaged for a total score. Internal consistency for this sample was excellent (Men: α = 0.91; Women: α = 0.89).

#### Outcome measures

To assess ED pathology, we utilized the ED-15 [35]. The ED-15 includes 10 attitudinal items and 5 eating behavior items. The attitudinal items included two subscales—Weight and Shape Concerns and Eating Concerns. Participants indicated on a 6-point Likert scale how often they engage in certain eating behaviors and attitudes over the past week, such as worrying about losing control over their eating. The total score is calculated by taking the mean of the 10 attitudinal items, with higher scores indicating more ED pathology. The ED-15 has strong test–retest reliability, strong concurrent validity related to the EDE-Q in nonclinical samples, and has demonstrated clinical utility [35]. The internal consistency in this sample was excellent (Men: α = 0.94; Women: α = 0.93).

To measure body dissatisfaction in female participants, we used the Body Satisfaction Questionnaire-16b [36]. This 16-item scale was derived from the original Body Satisfaction Questionnaire (BSQ; [37]) and asks about one’s body image, negative feelings, thoughts in relation to one’s body, and the actions one may take as a way of coping. Responses are recorded on a 6-point Likert scale, ranging from “never” to “always”; items are summed for a total score and higher scores indicate greater body dissatisfaction (α = 0.96).

Of note, the BSQ was originally designed for and validated in women and has yet to be formally validated in samples of men. Additionally, men tend to focus more on muscularity and leanness in relation to ideal body shape [38]. Because we wanted a scale that captured this and reflected the male body image literature, we used the Male Body Attitudes Scale—Revised to measure body dissatisfaction in men (MBAS-R; 39). The MBAS-R is derived from the 24-item Male Body Attitudes Scale [38]. The revised version is a 15-item questionnaire with a 7-item subscale of muscularity, 5-item body fat subscale, and a 3-item height subscale. Participants record how often they think about a body dissatisfied or satisfied statement on a 5-point Likert scale, ranging from “never” to “always.” This revised scale has demonstrated strong reliability and convergent validity with other body image measures [39]. Total scores are calculated using summation, with higher scores indicating greater body dissatisfaction. (α = 0.90).

To assess depressive symptoms, we used the 8-item Patient Health Questionnaire (PHQ-8; [40]). Research has demonstrated the PHQ-8 has similar scores, specificity, and screening accuracy for major depression as the PHQ-9; it also has strong construct and concurrent reliability [41, 42]. The PHQ-8 omits one question from the PHQ-9 that is typically used to identify suicidality that inquiries about thoughts of self-harm or thoughts about death. However, studies have demonstrated that this last item does not accurately assess suicide risk [42]. Thus, we chose the PHQ-8. Items are scored on a 4-point scale, with higher scores indicating increased severity of depressive symptoms. Standard cut off score to identify possible major depression is 10. Internal consistency for this sample was good (Men: α = 0.88; Women: α = 0.87).

To measure aging anxiety, we used the Anxiety about Aging Scale (AAS; 29). Since we are primarily concerned with the way people engage in talk about the physical aspects of aging, we only used the physical appearance subscale. This decision is further supported by the fact that the physical appearance subscale was the only subscale in the scale development study to significantly correlate with age [29]. This subscale consists of five statements about aging that one may think or believe about their aging, such as “I have never lied about my age in order to appear younger,” or “I have never dreaded looking old.” Participants respond using a 5-point Likert scale ranging from ‘strongly agree’ to ‘strongly disagree,’ with total scores derived from a summation of scale items (Men: α = 0.70; Women: α = 0.68).

We utilized the General Anxiety Scale to measure anxiety symptoms (GAD-7; [43]). This scale has demonstrated strong reliability, validity, and generalizability for detecting anxiety in the general population [44]. Using a 4-point Likert scale, participants reported how often in the past two weeks they may have been bothered by specific problems related to worry. Items are summed for a total score; scores ≥ 10 indicate a probable anxiety disorder (Men: α = 0.90; Women: α = 0.91).

We used the EUROHIS QOL 8-item index to assess QOL (EUROHIS-QOL 8; [45]). The EUROHIS-QOL 8 is an 8-item questionnaire that gauges one’s QOL in several domains, including psychological, physical, social, and environmental domains [46]. Each question uses an individualized 5-point Likert scale, with scale items summed for a total summary score. Higher scores indicate better QOL. This scale has demonstrated satisfactory convergent and discriminant validity across multiple countries and individuals of various health status [46]. Internal consistency for this sample was good (Men: α = 0.85; Women: α = 0.85).

### Data analyses

If participants completed less than 30% of the survey (which would mean they did not complete the negative body talk measures), then they were not included/were removed from analyses. For individual measures, we used imputation by imputing item means if less than 25% was missing from that measure, except for the PHQ-8 (measure instructions state that if more than one item is missing, the measure is not to be summed). After using these two techniques, 7 individuals were missing the ED-15 (the only other measure with similar incompletions was the PHQ-8, with missing data from 4 participants). These participants were included in all the analyses. It appears that this measure may have been the most sensitive and participants deliberately chose to skip it as it was located in the middle of the survey. Of note, this is still only 7 out of 778 participants (only 0.90% of the sample).

Previous research found a moderate correlation (*r* = 0.54; [25]) between fat talk and old talk, suggesting possible overlap in elements of old talk and fat talk that drive negative outcomes. Therefore, we investigated the extent that both forms of negative body talk contribute to negative mental health and QOL outcomes when examined in the same model and when interacting with age. We conducted multiple linear regression models to investigate each outcome; all five assumptions of multiple linear regressions were met (linear relationship, absence of multicollinearity, independence of observations, homoscedasticity, multivariate normality). Regression models included the continuous variables of fat talk, old talk, age, and the interactions of age with fat talk and age with old talk as predictors (x) of health outcomes (y). Analyses were divided into primary outcomes (ED pathology, body dissatisfaction, and depression) and secondary outcomes (aging anxiety, general anxiety, and QOL). Primary and secondary outcomes were identified utilizing the body dissatisfaction and negative body talk literature. Research has identified body dissatisfaction as a robust risk factor for ED pathology and depression (e.g., [6, 47–49]). Additionally, negative body talk is a well-established risk factor for body dissatisfaction (e.g., [19]). Thus, ED pathology, body dissatisfaction, and depression were chosen as primary outcomes and all other outcomes were secondary. In line with previous literature [25], we covaried for BMI in all models. All models were run separately for men and women to understand any similarities or differences between genders.

When a significant interaction was detected, age was partitioned into three age categories based on developmental life stages that also fell into approximately 20-year increments (18–40, 41–59, 60 +) to further investigate the nature of the interaction. While we initially sought to maintain the same age groups as Becker et al. ([25]; 18–29, 30–45, 46–60, 61 +), these categories did not maintain internal validity within our sample. Thus, we partitioned age into the three categories instead in an effort to balance internal validity with our data and external validity with developmental life stage. Within each age category, we conducted separate bivariate correlations to examine the relations between fat talk and/or old talk and the outcome variables. We then calculated Fisher r to z transformations to examine differences in the magnitude and/or directions of the correlations between fat talk and/or old talk and outcomes between each age category.

## Results

Before proceeding with our main analyses, we first examined data distributions and bivariate correlations between variables used in our models in both men and women because our analytic strategy separated men and women. See Tables 1, 2 and 3 for correlations and descriptive statistics. For men, fat talk significantly correlated with all variables examined except for age. The same was true for women with one exception; fat talk showed as small but significant negative correlation with age. With regards to old talk, for men all variables including age correlated with old talk. We found the same for women.

**Table 2:** Bivariate correlations for all variables in men

	Fat talk	Old talk	BMI	Age	ED path	BD	Depression	Age Anx	Gen. Anx	QOL
Fat Talk	–									
Old Talk	0.553^a^	–								
BMI	0.388^a^	0.265^a^	–							
Age	− 0.064	0.331^a^	0.323^a^	–						
ED Path	0.723^a^	0.437^a^	0.261^a^	− 0.199^a^	–					
BD	0.690^a^	0.377^a^	0.253^a^	− 0.209^a^	0.773^a^	–				
Depression	0.348^a^	0.168^b^	0.101	− 0.199^a^	0.501^a^	0.450^a^	–			
Age Anx	0.260^a^	0.411^a^	− 0.038	− 0.068	0.328^a^	0.303^a^	0.225^a^	–		
Gen. Anx	0.359^a^	0.156^b^	0.045	− 0.238^a^	0.481^a^	0.411^a^	0.732^a^	0.213^a^	–	
QOL	− 0.336^a^	− 0.188^a^	− 0.184^b^	0.061	− 0.384^a^	− 0.447^a^	− 0.682^a^	− 0.270^a^	− 0.535^a^	−

BMI = Body Mass Index; ED Path. = eating disorder pathology; BD = body dissatisfaction; Age Anx. = aging anxiety; Gen. Anx. = general anxiety; QOL = quality of life; ^a^*p* < 0.001; ^b^*p* < 0.01

**Table 3:** Bivariate correlations for all variables in women

	Fat talk	Old talk	BMI	Age	ED path	BD	Depression	Age Anx	Gen. Anx	QOL
Fat talk	–									
Old Talk	0.484^a^	–								
BMI	0.250^a^	0.122^b^	–							
Age	− 0.224^a^	0.241^a^	0.156^a^	–						
ED path	0.720^a^	0.395^a^	0.314^a^	− 0.240^a^	–					
BD	0.718^a^	0.386^a^	0.383^a^	− 0.227^a^	0.886^a^	–				
Depression	0.359^a^	0.199^a^	0.096^c^	− 0.356^a^	0.541^a^	0.543^a^	–			
Age Anx	0.236^a^	0.424^a^	− 0.046	− 0.014	0.331^a^	0.300^a^	0.145^a^	–		
Gen. Anx	0.277^a^	0.127^b^	0.008	− 0.340^a^	0.358^a^	0.350^a^	0.736^a^	0.135^b^	–	
QOL	− 0.300^a^	− 0.215^a^	− 0.275^a^	0.206^a^	− 0.465^a^	− 0.493^a^	− 0.688^a^	− 0.178^a^	− 0.500^a^	–

BMI = Body Mass Index; ED Path. = eating disorder pathology; BD = body dissatisfaction; Age Anx. = aging anxiety; Gen. Anx. = general anxiety; QOL = quality of life

^a^*p* < 0.001; ^b^*p* < 0.01; ^c^*p* < 0.05

### Primary outcomes

#### Men

**Fig. 1:**
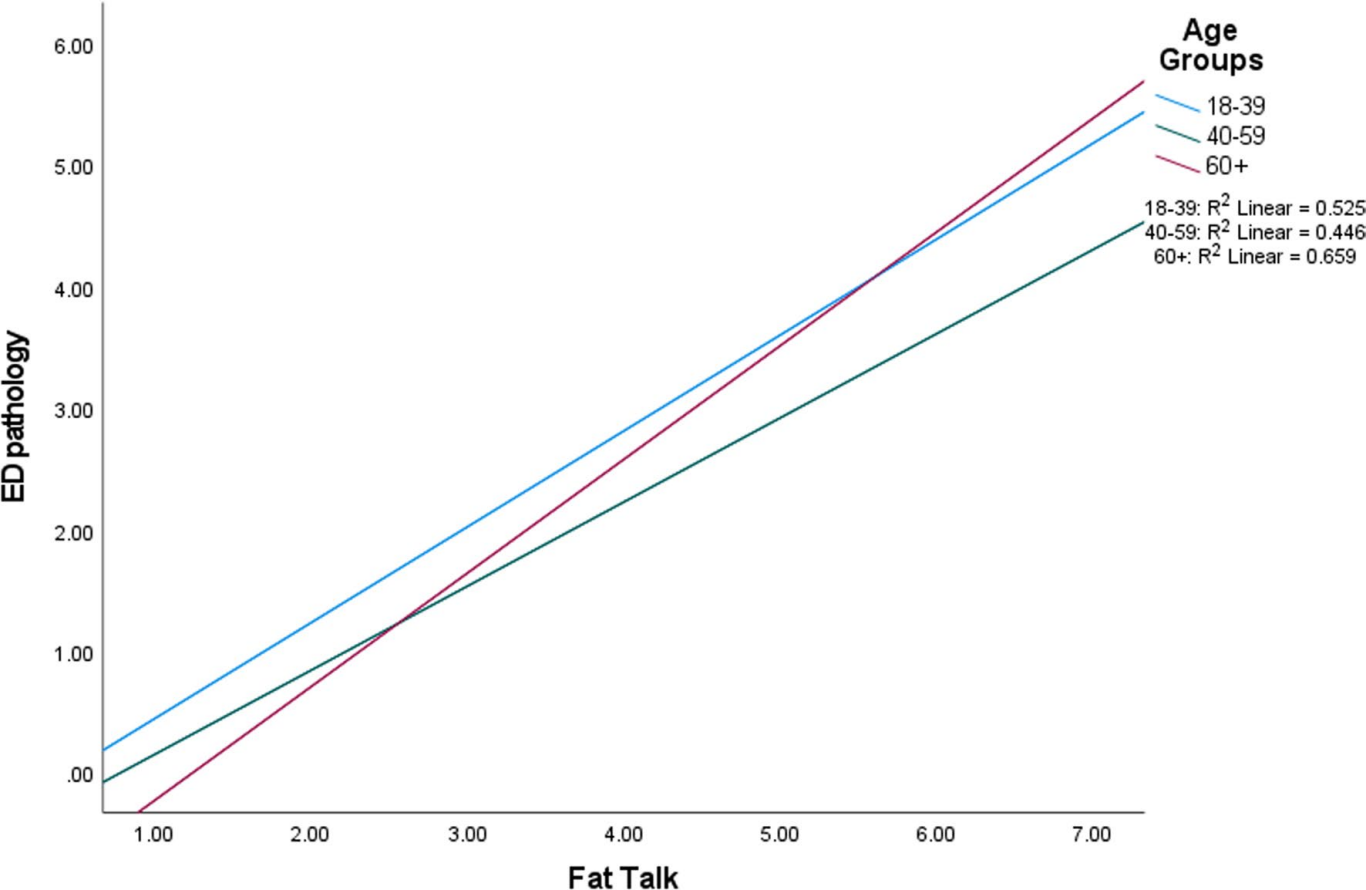
Interaction between fat talk and age in men, partitioned by age group

**Fig. 2:**
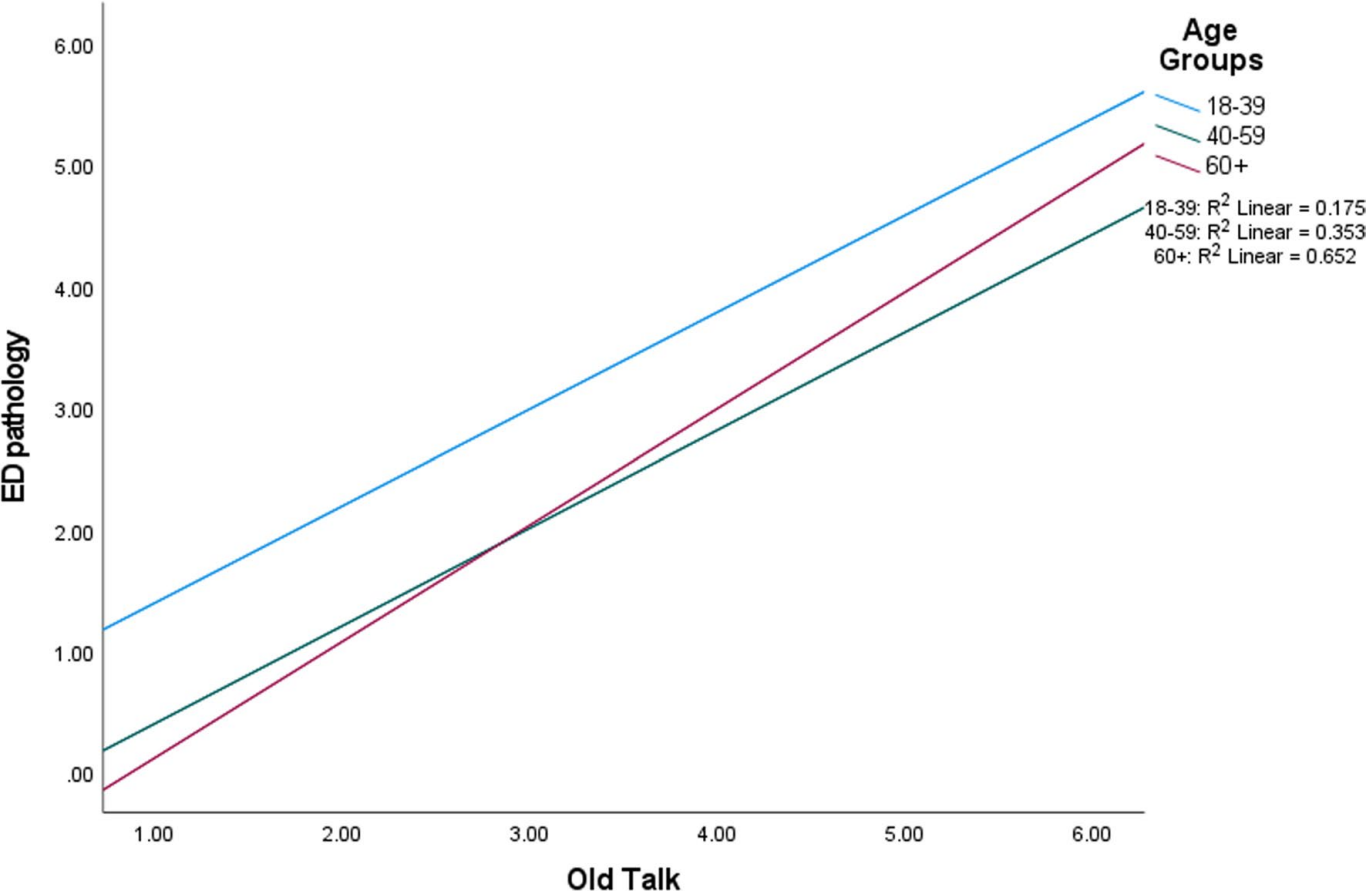
Interaction between old talk and age in men, partitioned by age group

Regarding ED pathology, there was a significant main effect for fat talk (*p* < 0.001) such that greater fat talk engagement was associated with greater ED pathology; there was no significant effect for old talk (Table 4). Results also indicated main effects for BMI and age, such that higher BMI (*p* = 0.026) was related to higher ED pathology and older age was associated with less ED pathology (*p* = 0.006). The effect size for the overall model was large in magnitude. Lastly, there were two significant interactions (Fat talk x Age, *p* < 0.001; Old talk x Age, *p* = 0.003) detected for men. When partitioned by age category to break down this interaction, Fisher r to z transformations revealed a significant difference in the magnitude of the fat talk and ED pathology correlations between age groups 40–59 and 60+; the correlation was stronger in the oldest age group (*p* = 0.04; Fig. 1). The correlations between old talk and ED pathology were different between the 18–40 and 60 + age group (*p* < 0.001) and the 40–59 and 60 + age group (*p* = 0.01), with the oldest age group indicating the strongest correlation (Fig. 2).

**Table 4:** Linear regression primary outcomes in men

Variables	*β*	*t*	95% CI	R^2^ change	Adj. R	*f*^2§^
*ED pathology*					0.57	1.38
BMI	0.11	2.25^c^	(0.004, 0.06)	0.07		
Fat Talk	0.82	7.97^a^	(6.79, 1.13)	0.46		
Old talk	− 0.14	− 1.14	(− 0.57, 0.15)	0.001		
Age	− 0.28	− 2.79^b^	(− 0.03, − 0.006)	0.04		
FTxAge	− 0.44	− 2.59^b^	(− 0.02, − 0.002)	0.001		
OTxAge	0.60	3.04^b^	(0.005, 0.02)	0.014		
*Body dissatisfaction*					0.52	1.11
BMI	0.09	1.71	(− 0.03, 0.43)	0.07		
Fat Talk	0.65	6.02^a^	(4.15, 8.18)	0.41		
Old talk	− 0.21	− 1.63	(− 6.09, 0.57)	0.00		
Age	− 0.44	− 4.08^a^	(− 0.40, − 0.14)	0.04		
FTxAge	− 0.11	− 0.60	(− 0.08, 0.04)	0.003		
OTxAge	0.52	2.51^c^	(0.02, 0.17)	0.01		
*Depression*					0.14	0.19
BMI	0.07	0.97	(− 0.07, 0.20)	0.01		
Fat Talk	0.39	2.69^b^	(0.43, 2.76)	0.11		
Old talk	− 0.03	− 0.18	(− 2.11, 1.76)	0.001		
Age	− 0.21	− 1.44	(− 0.13, 0.02)	0.03		
FTxAge	− 0.23	− 0.98	(− 0.05, 0.02)	0.001		
OTxAge	0.25	0.91	(− 0.02, 0.06)	0.003		

ED Pathology = eating disorder pathology; BMI = body mass index; FTxAge = fat talk and age interaction; OTxAge = old talk and age interaction

^**§**^Cohen’s *f*^2^ as estimate of effect size: 0.02 = small, 0.15 = medium, 0.35 = large

^a^*p* < 0.001; ^b^*p* = 0.01; ^c^*p* = 0.05

**Fig. 3:**
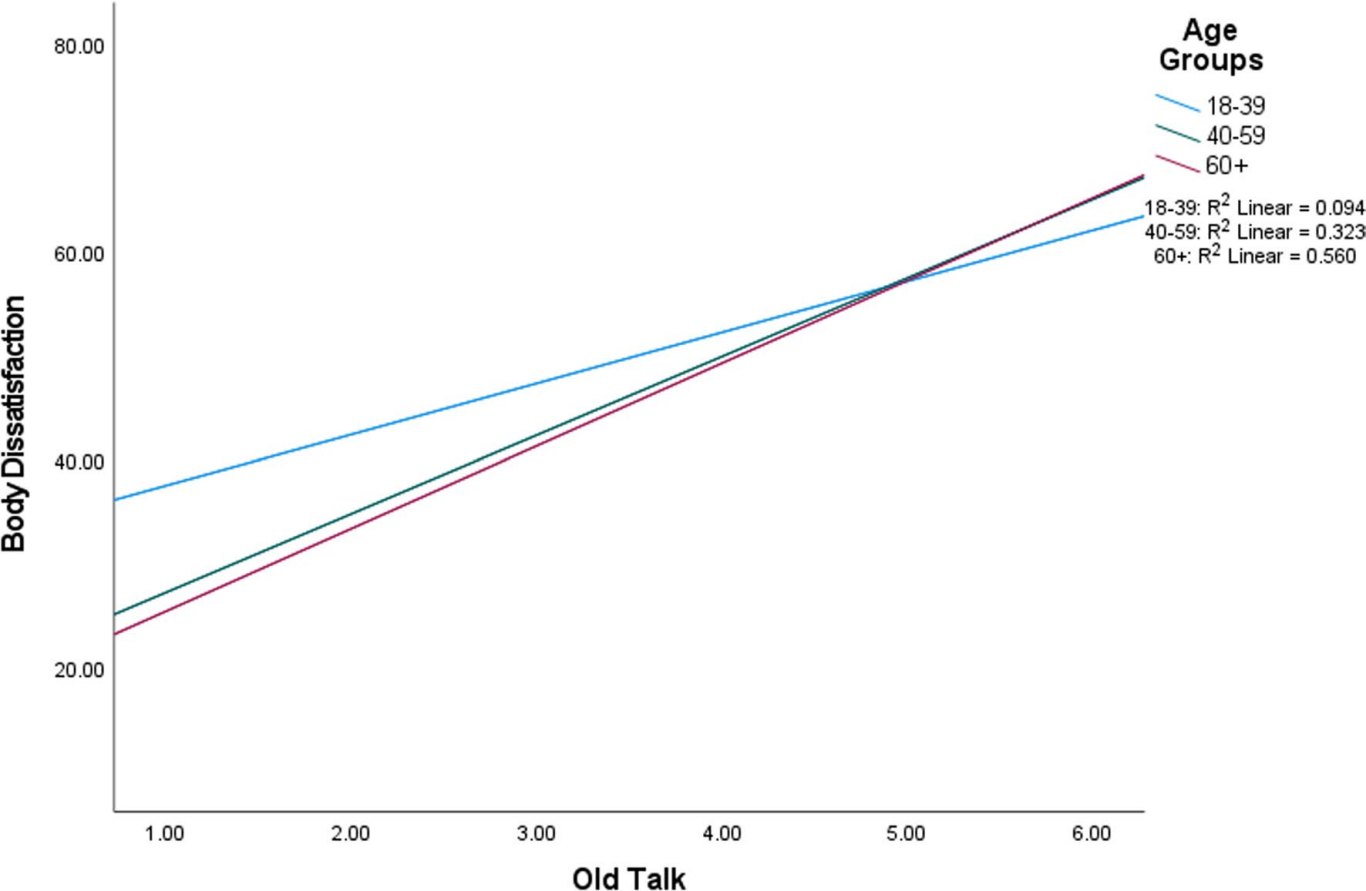
Interaction between old talk and body dissatisfaction in men, partitioned by age group

Regarding body dissatisfaction, significant main effects were found for fat talk and age. Engaging in more fat talk was associated with more body dissatisfaction (*p* < 0.001) and older age was associated with less body dissatisfaction (*p* < 0.001). No significant main effects were found for old talk. The effect size for the overall model was large in magnitude. There was one significant interaction between old talk and age (*p* = 0.013; Fig. 3). Further investigation indicated a significant difference in the correlations of old talk and body dissatisfaction between age groups 18–39 and 60+ (*p* < 0.001), and a difference between the two oldest age groups (*p* = 0.04); correlations were strongest in the 60+ group.

Regarding depressive symptoms, there was a main effect for fat talk frequency (*p* = 0.008), but no significant effects for old talk. More engagement in fat talk was associated with worse depression. No other significant main effects were found, and no interactions were detected. The effect size for the overall model was medium-to-large in magnitude.

#### Women

Firstly, there was a main effect for old talk (*p* = 0.05) and fat talk (*p* < 0.001) in relation to ED pathology in women, indicating greater engagement in each type of negative body talk was associated with greater ED pathology. Additionally, higher BMI (*p* < 0.001) was associated with greater ED pathology. There were no significant interactions (Table 5). The effect size for the overall model was large in magnitude.

**Table 5:** Linear regression primary outcomes in women

Variables	*β*	*t*	95% CI	R^2^ change	Adj. R	*f*^2§^
*ED pathology*					0.57	1.35
BMI	0.19	5.61^a^	(0.03, 0.05)	0.10		
Fat talk	0.66	7.22^a^	(0.50, 0.87)	0.44		
Old talk	0.21	1.95	(− 0.002, 0.68)	0.005		
Age	− 0.06	− 0.59	(− 0.02, 0.01)	0.03		
FTxAge	− 0.18	− 1.40	(− 0.009, 0.002)	0.003		
OTxAge	− 0.02	− 0.09	(− 0.008, 0.008)	0.00		
*Body dissatisfaction*					0.59	1.47
BMI	0.26	7.97^a^	(0.53, 0.88)	0.14		
Fat talk	0.67	7.52^a^	(6.77, 11.56)	0.42		
Old talk	0.04	0.42	(− 3.49, 5.36)	0.004		
Age	− 0.16	− 1.74	(− 0.38, 0.02)	0.02		
FTxAge	− 0.20	− 1.66	(− 0.13, 0.01)	0.001		
OTxAge	0.22	1.34	(− 0.03, 0.17)	0.002		
*Depression*					0.24	0.34
BMI	0.11	2.56^c^	(0.02, 0.15)	0.01		
Fat Talk	0.14	1.20	(− 0.35, 1.43)	0.11		
Old talk	0.24	1.71	(− 0.21, 3.09)	0.004		
Age	− 0.39	− 3.11^b^	(− 0.20, − 0.04)	0.13		
FTxAge	− 0.08	− 0.51	(− 0.03, 0.02)	0.00		
OTxAge	0.06	0.28	(− 0.03, 0.04)	0.00		

ED Pathology = eating disorder pathology; BMI = body mass index; FTxAge = fat talk and age interaction; OTxAge = old talk and age interaction

^**§**^Cohen’s *f*^2^ as estimate of effect size: 0.02 = small, 0.15 = medium, 0.35 = large

^a^*p* < 0.001; ^b^*p* = 0.01; ^c^*p* = 0.05

Regarding body dissatisfaction, again a main effect for fat talk was detected (*p* < 0.001), but no main effect for old talk was found. Greater engagement in fat talk was significantly associated with more body dissatisfaction, as was having a higher BMI (*p* < 0.001). No other significant main effects or any interactions were detected. The effect size for the overall model was large in magnitude.

Lastly, no main effects for fat talk or old talk in relation to depressive symptoms were found. However, main effects for BMI and age indicated that higher BMI was associated with greater depression (*p* = 0.011), while older age was associated with less depression (*p* = 0.002) among women. No significant interactions were detected. The effect size for the overall model was medium-to-large in magnitude.

### Secondary outcomes

#### Men

**Fig. 4:**
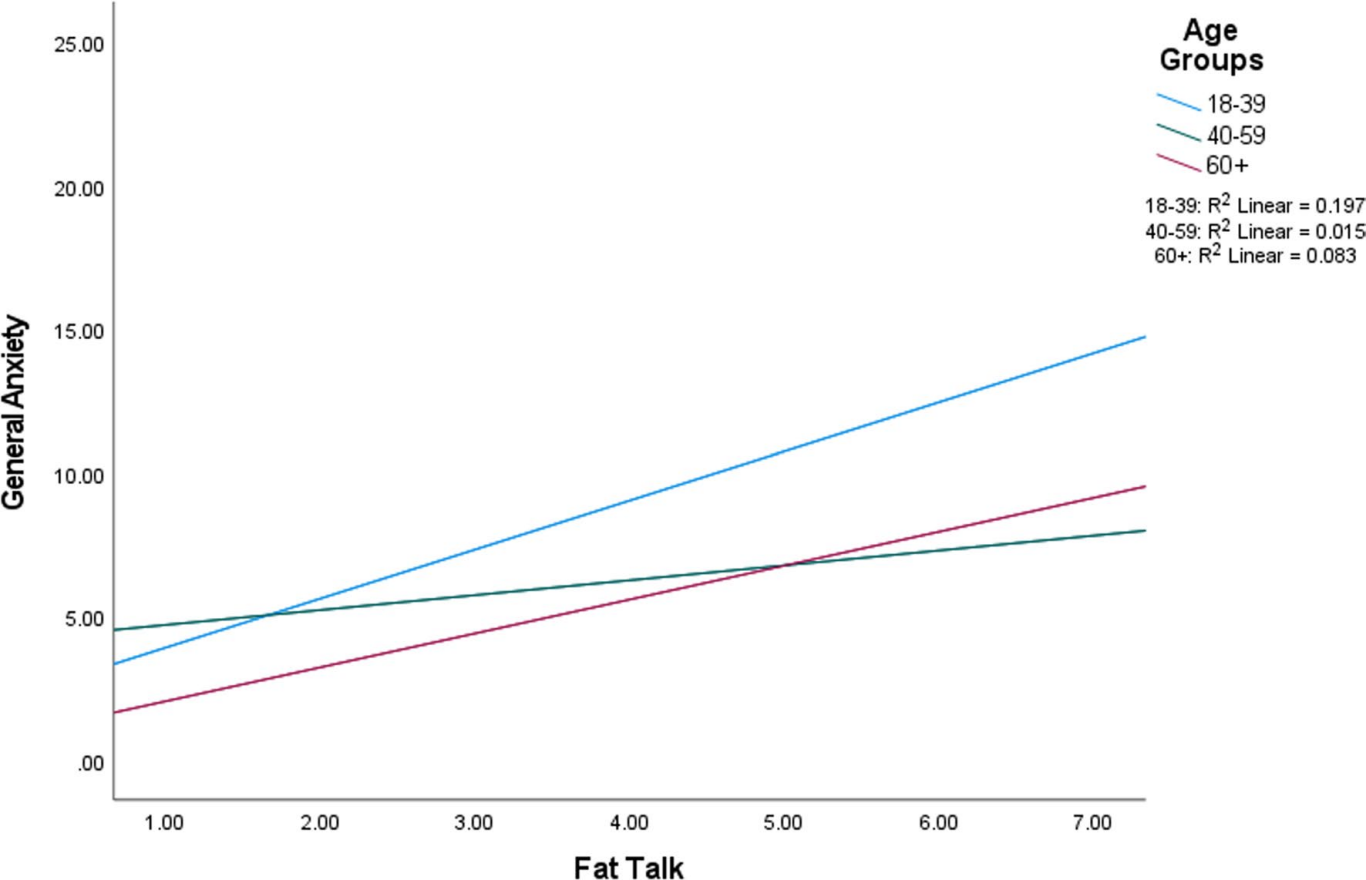
Interaction between fat talk and general anxiety in men, partitioned by age group

In the aging anxiety models, only a main effect for old talk was detected, with more old talk engagement associated with greater aging anxiety in men (*p* = 0.008). Regarding general anxiety, there was a main effect for fat talk and general anxiety (*p* < 0.001); greater engagement in fat talk was associated with greater anxiety. There was no main effect for old talk. There also was a significant interaction for fat talk and age (*p* = 0.033; Fig. 4). Fisher r to z transformations indicated a significant difference in fat talk and general anxiety correlations between the 18–39 and 40–59 age groups (*p* = 0.008); correlations indicated a stronger relationship in the younger age group. Lastly, while the overall model for QOL was significant for men (*p* < 0.001), no individual predictor variables were independently significant (Table 6). The effect sizes for all secondary outcome models for men were small-to-medium or medium-to-large in magnitude.

**Table 6:** Linear regression secondary outcomes in men

Variables	*β*	*t*	95% CI	R^2^ change	Adj. R	*f*^2§^
*Aging anxiety*					0.21	0.30
BMI	− 0.09	− 1.44	(− 0.19, 0.03)	0.001		
Fat talk	0.20	1.43	(− 0.27, 1.67)	0.09		
Old talk	0.43	2.66^b^	(0.55, 3.70)	0.11		
Age	− 0.09	− 0.62	(− 0.08, 0.04)	0.03		
FTxAge	− 0.34	− 1.49	(− 0.05, 0.007)	0.005		
OTxAge	0.21	0.78	(− 0.02, 0.05)	0.002		
*General anxiety*					0.17	0.19
BMI	0.009	0.13	(− 0.12, 0.14)	0.002		
Fat talk	0.57	4.07^a^	(1.20, 3.45)	0.14		
Old talk	− 0.04	− 0.22	(− 2.04, 1.63)	0.002		
Age	− 0.07	− 0.53	(− 0.09, 0.05)	0.04		
FTxAge	− 0.50	− 2.15^c^	(− 0.07, − 0.003)	0.01		
OTxAge	0.31	1.15	(− 0.02, 0.07)	0.004		
*Quality of life*					0.10	0.13
BMI	− 0.10	− 1.48	(− 0.26, 0.04)	0.03		
Fat Talk	− 0.26	− 1.76	(− 2.47, 0.14)	0.08		
Old talk	0.02	0.13	(− 1.99, 2.27)	0.00		
Age	0.15	1.01	(− 0.04, 0.13)	0.005		
FTxAge	− 0.006	− 0.03	(− 0.04, 0.04)	0.00		
OTxAge	− 0.10	− 0.35	(− 0.06, 0.04)	0.00		

ED Pathology = eating disorder pathology; BMI = body mass index; FTxAge = fat talk and age interaction; OTxAge = old talk and age interaction

^**§**^Cohen’s *f*^2^ as estimate of effect size: 0.02 = small, 0.15 = medium, 0.35 = large

^a^*p* < 0.001; ^b^*p* = 0.01; ^c^*p* = 0.05

#### Women

Regarding aging anxiety in women, there was a main effect for old talk, but not fat talk; greater engagement in old talk was associated with more aging anxiety (*p* < 0.001). For general anxiety, only age was significant as older age was associated with less anxiety (*p* = 0.002). Additionally, more engagement in fat talk (*p* = 0.046) and higher BMI (*p* < 0.001) were associated with poorer QOL in women; older age was associated with better QOL in women (*p* = 0.037). We found no significant interactions for age x fat talk or age x old talk for any secondary outcomes among women (Table 7). The effect sizes for all secondary outcome models for women were medium-to-large in magnitude.

**Table 7:** Linear regression secondary outcomes in women

Variables	*β*	*t*	95% CI	R^2^ Change	Adj. R	*f*^2§^
*Aging anxiety*					0.22	0.28
BMI	− 0.08	− 1.84	(− 0.11, 0.003)	0.002		
Fat talk	0.19	1.55	(− 0.16, 1.38)	0.08		
Old talk	0.49	3.45^a^	(1.08, 3.93)	0.13		
Age	0.07	0.55	(− 0.05, 0.08)	0.009		
FTxAge	− 0.26	− 1.57	(− 0.04, 0.004)	0.006		
OTxAge	0.01	0.07	(− 0.03, 0.03)	0.00		
*General anxiety*					0.17	0.21
BMI	0.03	0.58	(− 0.05, 0.09)	0.00		
Fat talk	0.07	0.53	(− 0.69, 1.20)	0.08		
Old talk	0.15	1.01	(− 0.85, 2.64)	0.00		
Age	− 0.41	− 3.17^b^	(− 0.21, − 0.05)	0.10		
FTxAge	0.02	0.13	(− 0.03, 0.03)	0.00		
OTxAge	0.05	0.23	(− 0.04, 0.05)	0.00		
*Quality of life*					0.20	0.27
BMI	− 0.29	− 6.42^a^	(− 0.31, − 0.16)	0.08		
Fat Talk	− 0.25	− 2.00^c^	(− 1.99, − 0.02)	0.07		
Old talk	− 0.07	− 0.48	(− 2.26, 1.37)	0.007		
Age	0.27	2.09^c^	(0.005, 0.17)	0.06		
FTxAge	0.27	1.60	(− 0.005, 0.05)	0.002		
OTxAge	− 0.31	− 1.39	(− 0.07, 0.01)	0.003		

ED Pathology = eating disorder pathology; BMI = body mass index; FTxAge = fat talk and age interaction; OTxAge = old talk and age interaction

^**§**^Cohen’s *f*^2^ as estimate of effect size: 0.02 = small, 0.15 = medium, 0.35 = large

^a^*p* < 0.001; ^b^*p* = 0.01; ^c^*p* = 0.05

## Discussion

The primary aim of this study was to examine the associations of engaging in fat talk and old talk with ED pathology, body dissatisfaction, depression, and other mental health and QOL indicators in adult men and women. We placed old talk and fat talk in the same model to examine if one is more related to poorer mental health and QOL. We investigated how age interacts with these relationships as well.

Regarding gender specific outcomes, fat talk was significantly associated with ED pathology, body dissatisfaction, depression, and general anxiety in men; old talk was not significantly related. However, old talk was significantly associated with aging anxiety across all ages while fat talk was not. While there is no literature examining old talk and aging anxiety in men, research does support the prevalence of aging anxiety across the male adult lifespan. For instance, one study found that being younger is associated with greater appearance-related aging anxiety in men [50]. Furthermore, older men have reported awareness of sociocultural pressures to age well; thus, these standards may produce aging anxiety and more old talk as men undergo the aging process [21]. These past findings in tandem with the current study suggest the young ideal in Western culture not only impacts women but is pervasive among men as well.

Our hypotheses that fat talk would be related to outcome variables regardless of age and that old talk would interact with age received only partial support. The correlation between fat talk and ED pathology in men was significantly larger in the oldest group as compared to the midlife age group, though no significant difference was detected between the oldest and youngest age groups. Additionally, for men 60+, the correlation between old talk and both ED pathology and body dissatisfaction was greater than for the two younger age groups.

In summary, it appears that in men 60+ there is a greater association of both fat talk and old talk with ED pathology, as well as old talk and body dissatisfaction as compared to younger ages. It is possible that older men who engage in old talk and fat talk are more attuned to the young muscular ideal and actively resist the aging process rather than accepting age-related changes. Therefore, negative body talk in older men may be more related to ED pathology than in younger men because of active resistance to the aging process.

Of note, there may be other sociodemographic factors that play a role in how age and negative body talk interact, such as marital status and education. However, it can be hard to disentangle these factors when examining the effect of age because many demographics are expected in certain life stages. For instance, our youngest age group is largely college aged. As would be expected, our two oldest age groups were more likely to be married and have a bachelor’s or graduate degree. Overall, there is little to no literature examining the impact of accepting the aging process—or the impact old talk—on ED pathology and body dissatisfaction in men, or how various aspects of life stage may influence these relationships. This is likely due to the historical eating disorders patient stereotype—young, thin, white, and at least moderately affluent girls. Because this stereotype shaped body image and eating disorders research for so long, this research on men of all ages remains lacking. Thus, relations between acceptance of the aging process, body image, negative body talk, and ED pathology in men are something to be explored in future research.

In women, fat talk and ED pathology, body dissatisfaction, and poorer QOL were all significantly associated regardless of age; of these outcomes, old talk was only significantly associated with ED pathology. While we did not anticipate old talk being non-significant, Becker and colleagues [25] found that fat talk was more strongly related to body image and ED pathology than old talk. Thus, these results align with previous research in suggesting fat talk is more related than old talk to greater ED pathology and poorer body image in women.

As with men, old talk was associated with greater aging anxiety in women; however, in contrast to our data for men, we found no age interaction for women. Becker et al. [25] also found that old talk was more strongly related to appearance-related aging anxiety than fat talk. In addition, other research indicates that appearance-related aging anxiety is related to body surveillance in both college-aged and midlife women; it also is related to engaging in anti-aging behaviors for midlife women [23, 24]. Currently it is unclear if old talk worsens aging anxiety, vice versa, or (perhaps most likely) they are reciprocally related. Longitudinal research will be needed to tease this apart. If old talk worsens aging anxiety, then we may be able to develop interventions targeting old talk that reduce aging anxiety for women as they grow older. Therefore, future research should continue to investigate the relationship between old talk, aging anxiety, and body image to determine whether old talk is a potential target for intervention to reduce aging anxiety and promote healthy body image as women age. Finally, age did not significantly affect the associations of negative body talk with ED pathology and body dissatisfaction for women. This is consistent with the literature demonstrating the pervasive nature of negative body talk and body dissatisfaction across the lifespan for women [1].

When comparing outcomes between genders, age appears to be more influential on negative body talk’s relations with ED pathology and body dissatisfaction in men than women. To some degree, this fits with the existing literature as research indicates that body dissatisfaction is higher in woman than men across the lifespan (ages 18–88) and is unaffected by age in women [51]; therefore, the lack of interaction between age and old talk as well as age and fat talk for women makes sense. With respect to men, we know much less about body image as men age. The limited research examining male body image with age indicates that older men are ambivalent/conflicted about appearance changes with age, expressing both body discontent and gratitude for health, and acceptance of the aging process [52, 53]. Older men have also expressed weight as a source of feeling physically unattractive or cited being happy with their weight as a source of body satisfaction [52, 53].

In younger men, muscular ideal internalization has been linked with body dissatisfaction and eating pathology (e.g., tripartite influence model in men; [54]). Extrapolating from data in younger men, one potential explanation for the difference in age effects seen between men and women in this sample could be that age-related muscle loss, which occurs in both men and women, has a salience for men in relation to body dissatisfaction that it does not for women. Body image for men is more centered on the muscular ideal [55] versus the thin ideal for women, which is consistent across the lifespan. Because men naturally lose muscle with age, older men may experience elevated levels of muscle dissatisfaction, which may strengthen the relationship between negative body talk and body dissatisfaction as men age. Evidently, much remains unknown about how negative body talk and body image shift with aging in men and in what ways this shift may look different compared to women. The findings from the current study suggest that more research is needed to tease apart what may be a more complicated relationship between body image, ED pathology and negative body talk in men.

While we anticipated old talk and fat talk to be significantly associated with all outcome variables in both men and women, fat talk was significantly associated with several mental health outcomes while old talk was not. These results suggest that when evaluated together, fat talk may be primarily responsible for harm to body image and mental health. However, in Becker et al. [25], engagement in old talk correlated with ED pathology and body dissatisfaction independent of fat talk; old talk also significantly correlated with all outcome variables in the current study. Additionally, fat talk and old talk were moderately correlated (men: *r* = 0.553; women: *r* = 0.484). Thus, it is possible the overlapping elements of old talk and fat talk are what drive the associations with poor mental health outcomes and QOL. It may be that old talk is not entirely unrelated to mental health and QOL or is not harmful, but rather what matters regarding harm to mental health is what overlaps in the constructs of old talk and fat talk. However, because so few studies have examined old talk alone or in tandem with fat talk, it is difficult to interpret these findings with confidence. Furthermore, we created the old talk questionnaire by modifying the same assessment (NBTS) used to measure fat talk. It is possible the overlap between the constructs may be partially due to using such similar measures. However, the correlation between the two constructs in this study was almost identical to Becker et al.’s findings, despite using different assessments for both forms of negative body talk. Thus, research should continue investigation of the relationship between old talk and fat talk; whether old talk is a phenomenon to target to improve body image independently of fat talk is still unknown.

There are several limitations to the present research that are worth noting. First, we did not use a validated measure to assess old talk engagement. Though the internal consistency was excellent for our old talk measure in both men and women in this sample, men may engage in old talk differently than women. Future research should investigate if there are any gender differences in the way adults engage in old talk and explore creating validated instruments for measuring old talk engagement in both genders. To assess fat talk, we modified the NBTS to be gender neutral and this measure was not previously validated in men. The questionnaires were also presented to participants in the same order and were not counter-balanced, thus it is possible results were influenced by order effects.

Additionally, while a strength of this study was the inclusion of men as old talk had yet to be evaluated in this population, we did not sample enough gender minority individuals to include in our analyses. Old talk has yet to be examined in individuals of gender minorities and should be included in future research exploring age-related body talk. Furthermore, perceptions of aging differ between cultures and levels of socioeconomic status [56]. Various perceptions of aging may affect engagement in old talk and its relation to mental health. Some Western cultures view aging negatively and socially devalue individuals in older age [56]. This negative perception of aging may increase resistance to aging-related appearance changes and increase old talk engagement. In contrast, if a culture views aging more favorably, old talk may be less common and less related to mental health. Higher socioeconomic status is also related to more negative views of aging [56]. Therefore, it is important to note that this is a Western sample with a higher socioeconomic status; thus, future research should explore old talk and its relation to poor mental health outcomes in more diverse samples.

This study was also a cross-sectional online study, thus no causality between relationships could be determined. As noted above, the relationship between old talk and mental health and QOL outcomes need to be investigated longitudinally, both independently of fat talk and together to better understand the potential harm of engaging in old talk. Finally, recruitment occurred primarily during the COVID-19 pandemic. Recent research suggests body image and ED pathology worsened during the pandemic, alongside depression and anxiety (e.g., [57–58]). Thus, it is possible that mental health severity in this sample was heightened by the pandemic, which in turn could also influence the relationship between negative body talk and mental health.

## Conclusions

Overall, while old talk is significantly correlated with an array of mental health and QOL measures, fat talk appears to be more influential in these relationships. However, old talk is still significantly understudied. Much remains unknown about how it operates alongside and overlaps with fat talk in influencing body image and other aspects of mental health across the lifespan. Gaining a better understanding of this phenomenon may improve our understanding of how to combat the young-ideal in Western society and how to promote healthy body image and mental health, especially as adults enter midlife and beyond.

## Data Availability

The dataset generated and/or analyzed during the current study is not publicly available but is available from the corresponding author on reasonable request.

## Appendix

*Modified Old Talk Scale (Negative Body Talk Scale Modified for Old Talk)*

When talking with your friends, how often do you say things like…

(Participants responded on Likert scale of 1–7, Never to Always).

I wish my body looked as young as theirs.

I need to look younger.

I feel old.

If it weren’t for my age I’d have a better looking stomach like theirs.

I'm too old for this style of clothing.

Why can’t my body look as young as theirs?

They have such a perfect, young body.

I need to start watching for wrinkles.

They look so good for their age.

I wish I was younger.

I wish my skin looked as youthful as theirs.

I think I look old.

You never have to worry about looking old.

*Original Negative Body Talk Scale*

When talking with your friends, how often do you say things like…

(Participants responded on Likert scale of 1–7, Never to Always).

I wish my body looked like hers.

I need to go on a diet.

I feel fat.

She has a perfect stomach.

This outfit makes me look fat.

Why can’t my body look like hers?

She has a perfect body.

I need to start watching what I eat.

She’s in such good shape.

I wish I was thinner.

I wish my abs looked like hers.

I think I’m getting fat.

You never have to worry about gaining weight.

## Abbreviations

ED: Eating disorder
QOL: Quality of life
BMI: Body Mass Index
NBTS: Negative Body Talk Scale
BSQ: Body Satisfaction Questionnaire
MBAS-R: Male Body Attitudes Scale Revised
PHQ-8: 8-Item Patient Health Questionnaire
PHQ-9: 9-Item Patient Health Questionnaire
AAS: Anxiety about Aging Scale
GAD: General Anxiety Scale
COVID-19: Coronavirus disease of 2019

## Footnotes

1 We identified six participants located outside of the US based on participants reporting international currency in their income reporting (which was inconsistently completed by participants, so is not reported in this paper). International breakdown: United Kingdom: n = 2, European Union: n = 4.

2 Based on variable type (e.g., continuous, categorical, and ordinal), we conducted a one-way ANOVA to examine differences in age and BMI, a Kruskall–Wallace test for education, and a chi-square test for marital status.
